# TC10 Is Regulated by Caveolin in 3T3-L1 Adipocytes

**DOI:** 10.1371/journal.pone.0042451

**Published:** 2012-08-10

**Authors:** Dave Bridges, Louise Chang, Irfan J. Lodhi, Natalie A. Clark, Alan R. Saltiel

**Affiliations:** Life Sciences Institute, University of Michigan, Ann Arbor, Michigan, United States of America; Institut Curie, France

## Abstract

**Background:**

TC10 is a small GTPase found in lipid raft microdomains of adipocytes. The protein undergoes activation in response to insulin, and plays a key role in the regulation of glucose uptake by the hormone.

**Methodology/Principal Findings:**

TC10 requires high concentrations of magnesium in order to stabilize guanine nucleotide binding. Kinetic analysis of this process revealed that magnesium acutely decreased the nucleotide release and exchange rates of TC10, suggesting that the G protein may behave as a rapidly exchanging, and therefore active protein *in vivo*. However, in adipocytes, the activity of TC10 is not constitutive, indicating that mechanisms must exist to maintain the G protein in a low activity state in untreated cells. Thus, we searched for proteins that might bind to and stabilize TC10 in the inactive state. We found that Caveolin interacts with TC10 only when GDP-bound and stabilizes GDP binding. Moreover, knockdown of Caveolin 1 in 3T3-L1 adipocytes increased the basal activity state of TC10.

**Conclusions/Significance:**

Together these data suggest that TC10 is intrinsically active *in vivo,* but is maintained in the inactive state by binding to Caveolin 1 in 3T3-L1 adipocytes under basal conditions, permitting its activation by insulin.

## Introduction

TC10 is a Rho family GTPase, most similar in primary sequence and structure to Cdc42 and TC10β or TCL. This protein is expressed in a wide variety of tissues, but its role is best studied in adipocytes. Fractionation studies indicated that TC10 is present in lipid raft microdomains, and immunohistochemical studies indicate the presence of the protein in caveolar rosette structures in adipocytes [1], [2]. TC10 is activated in response to insulin in a CAP-dependent process [3]. Once activated, it binds to several effector proteins including CIP4 [4], [5], Exo70 [6], [7] and Par6B [8]. Dominant-negative and knock down experiments indicate that TC10 plays a critical role in insulin-stimulated GLUT4 translocation and glucose uptake [1]–[3], [9].

The activation state of small GTPases depends on whether GDP or GTP is bound. While the GDP-bound form is inactive, active GTP-bound GTPases bind effector proteins. These nucleotides normally exchange slowly, a process that can be accelerated by guanine nucleotide exchange factors (GEFs; reviewed in [10]–[13]). Therefore, GEFs are required for activation of most small GTPases. On the other hand, some GTPases bind nucleotides with weaker affinity, exchanging with free nucleotides more rapidly. These proteins require guanine nucleotide dissociation factors (GDIs; reviewed in [14], [15]) to maintain the GDP-bound, inactive state. A third class of regulatory proteins named GTPase activating proteins stimulate GTP hydrolysis and promote G protein inactivation (GAPs; reviewed in [12], [13]).

All small GTPases require magnesium as a nucleotide binding cofactor. This ion is normally bound with high affinity and assists in the co-ordination of the G protein with the beta and gamma phosphates of the guanine nucleotide [16]. Excess magnesium is not required to stabilize nucleotide binding for most GTPases. *In vitro* effector binding experiments with TC10 suggested that supplemental magnesium was required to stabilize TC10-effector complexes [17]. Therefore we investigated the role of magnesium in nucleotide binding using bacterially expressed TC10.

We found that TC10 has an atypical requirement for high concentrations of magnesium in order to stably bind guanine nucleotides. Furthermore, the high exchange rate of TC10 produces the constitutive activation of this protein. Finally, we found that direct association of TC10 with Caveolin 1 stabilized the nucleotide-bound state and keeps TC10 inactive in adipocytes.

## Results

### TC10 Nucleotide Binding is Mg-dependent

**Figure 1:**
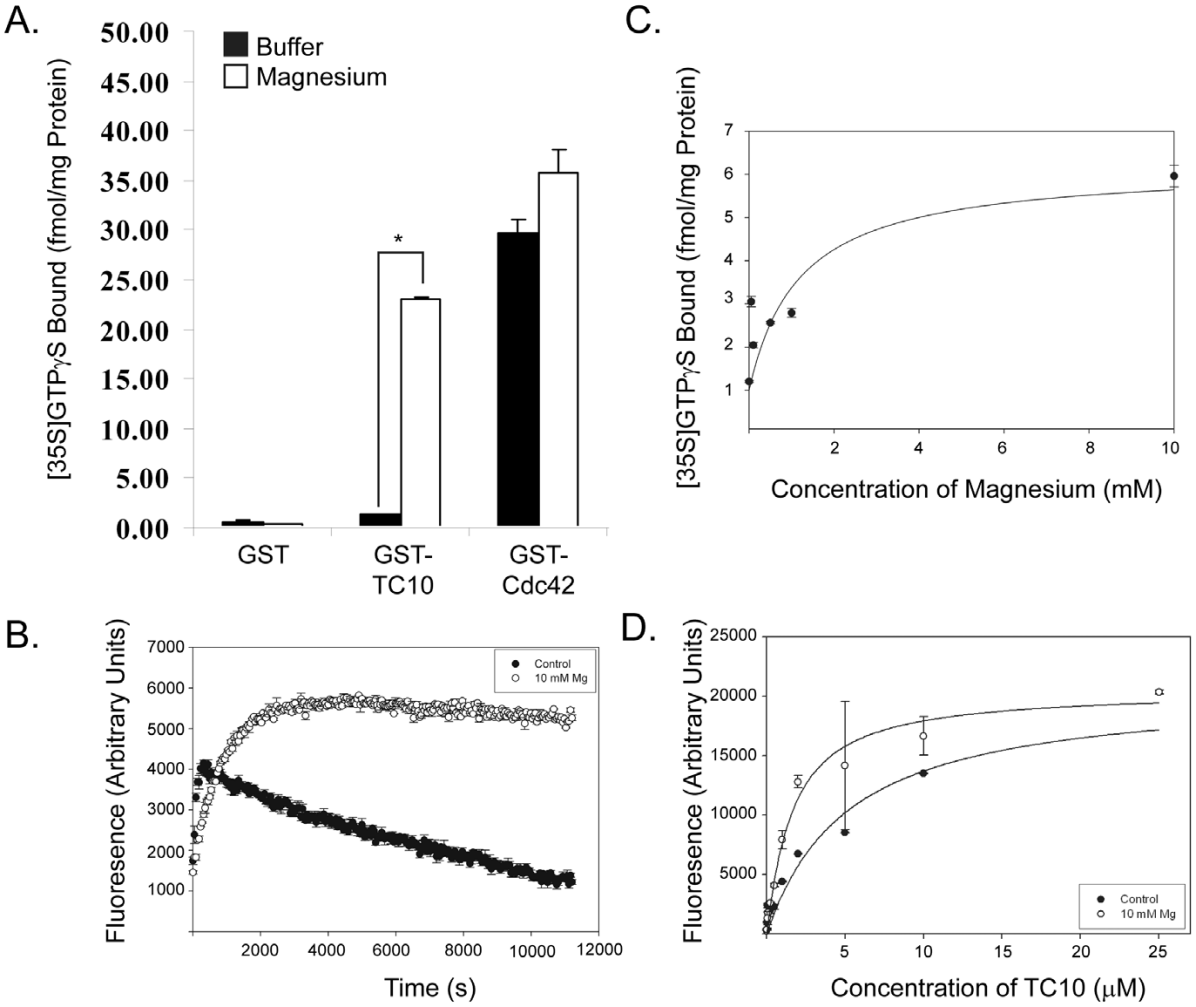
TC10 requires magnesium for stable nucleotide binding. A) Binding of [^35^S]-GTPγS to GST, GST-TC10 and GST-Cdc42 in the absence (black bars) or presence (white bars) of 10 mM MgCl_2_. B) Time-course of mantGDP binding to TC10 in the absence (control) and presence of 10 mM MgCl_2_. C) GST-TC10 was combined with [^35^S]-GTPγS in the presence of varying concentrations of supplemental magnesium. D) Titration of 50 nM mGDP with varying concentrations of GST-TC10 in the absence (control) or presence of 10 mM MgCl_2_. Samples were incubated 2 h at room temperature before fluorescence measurement. Curve fitting and K_D_ calculations were determined according to (20). Data is presented as mean with standard error bars. Asterisks indicate p<0.05.

Effector binding experiments with TC10 have previously shown that magnesium is required to maintain the activation state of TC10 [17], [18]. To study this requirement at the nucleotide binding level, we incubated radiolabeled GTPγS with recombinant GST, GST-TC10 or GST-Cdc42 in either the absence or presence of 10 mM magnesium chloride. These proteins were used directly as purified from bacterial lysates and therefore are already bound with endogenous GDP. Cdc42 was able to bind the nucleotide in both cases; whereas TC10 exhibited a strict requirement for supplemental Mg ions (Figure 1A). EDTA was not present in the purification of GST-fusion proteins, so the control level of nucleotide binding is likely due to trace magnesium purified along with the proteins.

In order to understand TC10’s equilibrium magnesium cofactor requirement, we used the fluorescent nucleotide analog mantGDP, which increases in fluorescence when bound by a protein. As Figure 1B shows, in the absence of magnesium, the fluorescent signal rapidly increases over time, but then decreases to near basal levels. In contrast, the presence of supplemental magnesium stabilizes the fluorescent signal after binding, indicating that supplemental magnesium is required by TC10 for nucleotide stability.

To examine the effects of magnesium on nucleotide binding, we varied the concentration of supplemental magnesium and monitored binding of radiolabelled GTPγS to GST-TC10. We found that high levels of magnesium (∼10 mM) are necessary for stabilization of nucleotide binding (Figure 1C). Equilibrium binding experiments revealed that the dissociation constant for mantGDP binding to nucleotide-free TC10 was 4.80+/−0.50 nM in the absence of magnesium and 1.38+/−0.23 nM in the presence of 10 mM MgCl_2_ (Figure 1D). In contrast, similar studies on Cdc42 showed that magnesium caused a decrease in affinity (from 0.76+/−0.21 nM in the absence to 2.08+/−0.23 nM in the presence of 10 mM MgCl_2_).

**Figure 2:**
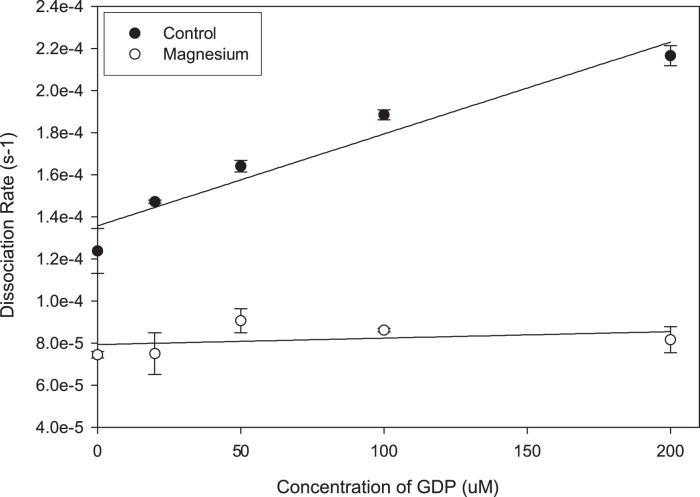
TC10 nucleotide exchange is inhibited by 10 mM **MgCl_2_.** TC10-mantGDP complexes were prepared in the presence of magnesium and then exchanged into buffer with or without 10 mM MgCl_2_. Release rates were determined from exponential decay curves and plotted versus the concentration of free GTP. Data are presented as mean with standard error bars.

Since the magnesium binding requirement appeared to be related to dissociation of the nucleotide, we performed an experiment in which nucleotide-free TC10 was bound to mantGDP in the presence of 10 mM MgCl_2_, and then exchanged into buffer either with or without magnesium by rapid gel filtration. The decrease in fluorescence, which correlates with the release of mantGDP from the GTPase, was then monitored and rates were calculated by first order rate equations. In the absence of supplemental magnesium or free guanine nucleotide, the nucleotide release rate was 1.36×10^−5^ s^−1^, compared with 0.79×10^−5^ s^−1^ in the presence of magnesium, a 1.7-fold increase (Figure 2). Nucleotide release is accelerated in the presence of free GTP, giving a measure of nucleotide exchange. Measurement of the nucleotide release rate in the presence of increasing concentrations of free GTP resulted in a modest increase in the GDP release rate in the presence of magnesium (a 1.8-fold increase at 200 µM and a calculated k_exchange_ of 0.031 M^−1^ s^−1^ Figure 2). In the absence of supplemental magnesium, we found that GTP potentiated nucleotide release more effectively (a 7.4-fold increase at 200 µM and a calculated k_exchange_ of 0.43 M^−1^ s^−1^, Figure 2 **)**. These data suggest that TC10 requires supplemental magnesium to stabilize nucleotides (either GDP or GTP) thereby greatly inhibiting the nucleotide exchange rate *in vitro.*

### TC10 has an Aberrant Magnesium Binding Site

**Figure 3:**
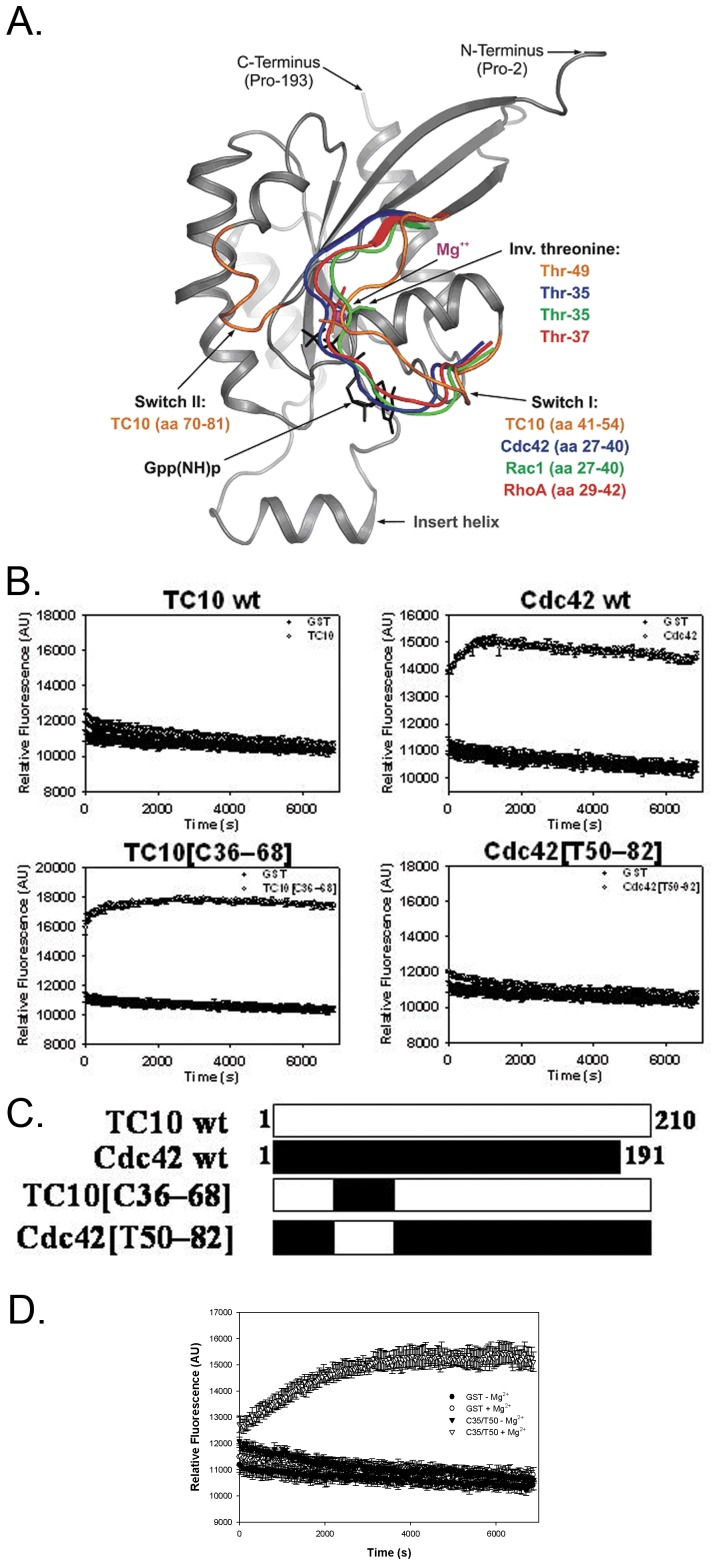
TC10 and Cdc42 are structurally divergent in the magnesium binding pocket. A) Structural comparison of TC10 with Cdc42, Rac1 and RhoA. The structure of the C-terminal truncated, active GppNHp-bound TC10 (PDB code 2ATX) is shown, which mainly differs in the conformation of the Switch I region (orange) as superimposed on the Switch regions of Cdc42 (1NF3; blue), Rac1 (1MH1; green) and RhoA (1A2B; red). Switch II, insert helix, GppNHp (a non-hydrolyzable GTP analog) and the respective invariant threonine of in the Switch I are highlighted. The latter normally contact the magnesium ion (sphere magenta), but this is not the case for TC10. B) Binding of mantGDP to TC10/Cdc42 chimeras (solid circles) in the absence of magnesium compared with a GST control (empty circles). Values shown are mean with standard error bars. C) Schematic of chimeras of TC10 and Cdc42 with black bars indicating Cdc42 and white bars indicating TC10. D) Rescue of mantGDP binding of the Cdc42[T50-82] construct with magnesium. The Cdc42[T50-82] construct was incubated with 10 mM magnesium and mantGDP binding assays were performed as described in panel B.

One hypothesis to explain the atypical magnesium requirement for nucleotide binding in TC10 is that the magnesium binding site is structurally distinct from that of other small G proteins (Figure 3A). Based on primary sequence, all residues that are expected to coordinate magnesium are conserved between TC10 and other GTPases. We thus examined the structures of TC10 (24) and other GTPases around their metal binding site. Although there were few differences in primary sequence, substantial structural divergence was noted in the metal binding region. In the GTP-bound state, the loop containing this residue diverged from the structure of Cdc42 by a root mean square deviation of 4.06 A?? angstroms compared with divergence of 1.47 A?? for the entire structural alignment (backbone atoms with the loop defined as Phe28 to Asn39; see Figure 3A).

For most GTPases a conformational change occurs in both the Switch I and Switch II regions upon displacing GDP with GTP. Structural analysis of TC10 indicated that while the Switch II region moves in the expected manner, the Switch I region remains locked in the GDP-like conformation when GTP is bound (Figure 3A).

We next examined TC10/Cdc42 chimeras in order to identify amino acid regions from Cdc42 that might explain the magnesium dependence of stable nucleotide binding for TC10. Chimeras were generated in which regions of TC10 were replaced with the corresponding region of Cdc42 (Figure 3B, schematic in Figure 3C). A chimera in which residues 50–82 of TC10 were replaced by residues 36–68 of Cdc42 displayed Mg-sensitive binding characteristic of TC10. This region, which lies between the Switch I and Switch II regions, comprises the ACK binding domain of the G protein (18). Therefore, it is likely that the amino acids 50–82 in TC10 plays a role in the Switch I mobility defect of TC10. To test this directly, we tested the ability of the Cdc42[T50–82] chimera to bind the GDP analog in the presence of magnesium. As shown in Figure 3D, this region of TC10 now confers magnesium sensitivity to the nucleotide binding ability of Cdc42.

### TC10 is Stabilized in the GDP-bound State by Directly Interacting with Caveolin 1

**Figure 4:**
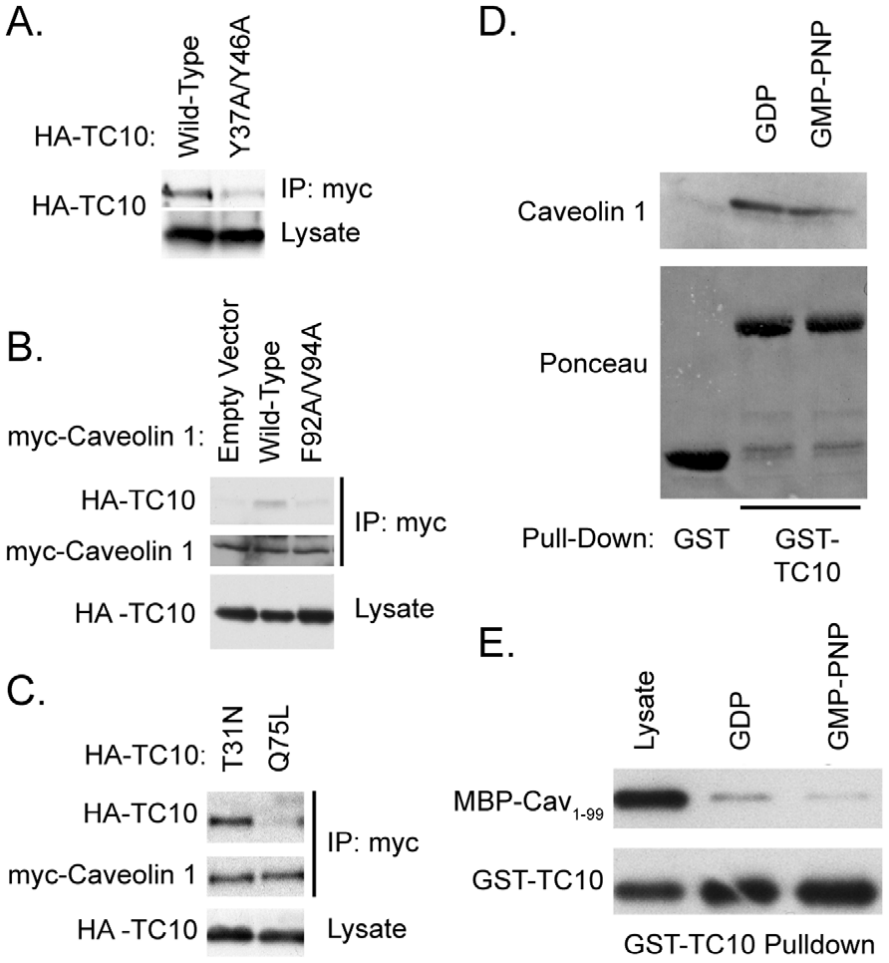
TC10 and Caveolin interact in a GDP-dependent manner. A) COS-1 cells were transfected with 100 ng of pKMyc-Caveolin 1 and 100 ng of pKH3-TC10 wild-type or pKH3-TC10 Y37A/Y46A. Lysates were immunoprecipitated with anti-myc antibodies and blotted for the HA epitope. B) COS-1 cells were transfected with pKMyc-Caveolin 1 wild-type or F92A/V94A alleles along with HA-TC10. Cells were lysed, and lysates were immunoprecipitated with anti-myc antibodies and blotted with either anti-myc or anti-HA antibodies. C) COS-1 cells were transfected with pKMyc-Caveolin 1 and pKH3-TC10 T31N or Q75L mutants. Lysates were immunoprecipitated with myc antibodies and blotted with either anti-myc or anti-HA. D) GST-TC10 bound to glutathione-agarose beads was preincubated with nucleotides as indicated, then used to precipitate proteins from a 3T3-L1 adipocyte lysate. Eluted fractions were probed with either anti-Caveolin antibodies or stained with Ponceau S. E) Purified MBP-Caveolin 1_(1–99)_ and GST-TC10 were incubated with the indicated nucleotide overnight at 4°C. Complexes were then purified by adding glutathione agarose for 1 h, followed by washing and elution in SDS sample buffer. Loaded and precipitated proteins were analyzed by blotting with anti-GST or anti-MBP antibodies.

It has been reported that Caveolin 1 behaves as a guanine dissociation inhibitor (GDI) for Cdc42 [19]. Based on sequence similarity of TC10 with Cdc42, as well as co-localization of TC10 with Caveolin 1 [1], we investigated whether Caveolin 1 could function as a GDI for TC10 in adipocytes. First we examined whether TC10 could form a direct interaction with Caveolin 1. Residues 37–46 of human TC10 (YANDAFPEEY) fits a consensus Caveolin binding motif (ΦXXXXΦXXΦ) present in several proteins [20]. Myc-Caveolin and HA-TC10 were co-transfected into COS-1 cells, and tested for association by co-immunoprecipitation. Figure 4A shows that wild-type TC10 is effectively co-immunoprecipitated with myc-Caveolin 1. To test whether mutation of these tyrosine residues is able to affect the Caveolin 1-TC10 interaction, we made tyrosine to alanine mutants, similar to those described for analogous Caveolin to Caveolin binding protein interactions [20]–[22]. Mutation of both tyrosines 37 and 46 in TC10 to alanine decreased the co-immunoprecipitation of Caveolin 1 with TC10 by greater than 75%. To test the role of the scaffolding domain of Caveolin 1, we mutated two key residues in Caveolin 1, Phe92 and Val94 into alanine residues. These residues have been shown previously to reduce Caveolin-target protein interactions [19], [20], [23], [24]. As shown in Figure 4B, mutagenesis of these residues decreased the amount of HA-TC10 which is co-immunoprecipitated with myc-Caveolin 1 by more than 85%.

Next, we looked at the ability of myc-Caveolin to co-precipitate activation state mutants of TC10. Figure 4C shows that the dominant-interfering mutant TC10-T^31^N was efficiently precipitated by Caveolin, while the constitutively active Q^75^L mutant was not. The interaction with the dominant negative allele of TC10 with Caveolin was more than ten-fold greater than that of the constitutively active allele. In order to verify that these mutants function by mimicking the GDP/GTP state of the protein, we pre-incubated GST-TC10 beads with GDP or a nonhydrolyzable GTP analog, and then used these to precipitate proteins from a 3T3-L1 adipocyte lysate. As Figure 4D shows, GST-TC10 was able to efficiently precipitate Caveolin 1, and this interaction was enhanced approximately two-fold after GDP binding. In order to examine the interaction of these proteins with purified proteins, we used an MBP-Caveolin 1 fusion which contained the first 99 amino acids of Caveolin. We co-incubated GST-TC10 and MBP-Caveolin1_(1–99)_ in the presence of GDP or a nonhydrolyzable GTP analog. We then purified complexes using glutathione-sepharose (Figure 4E). TC10-GDP precipitated more than four-fold more with MBP-Caveolin1_(1–99)_ than did the GTP analog bound TC10. These data suggest that TC10 interacts with Caveolin 1 more tightly in the presence of GDP than GTP.

**Figure 5:**
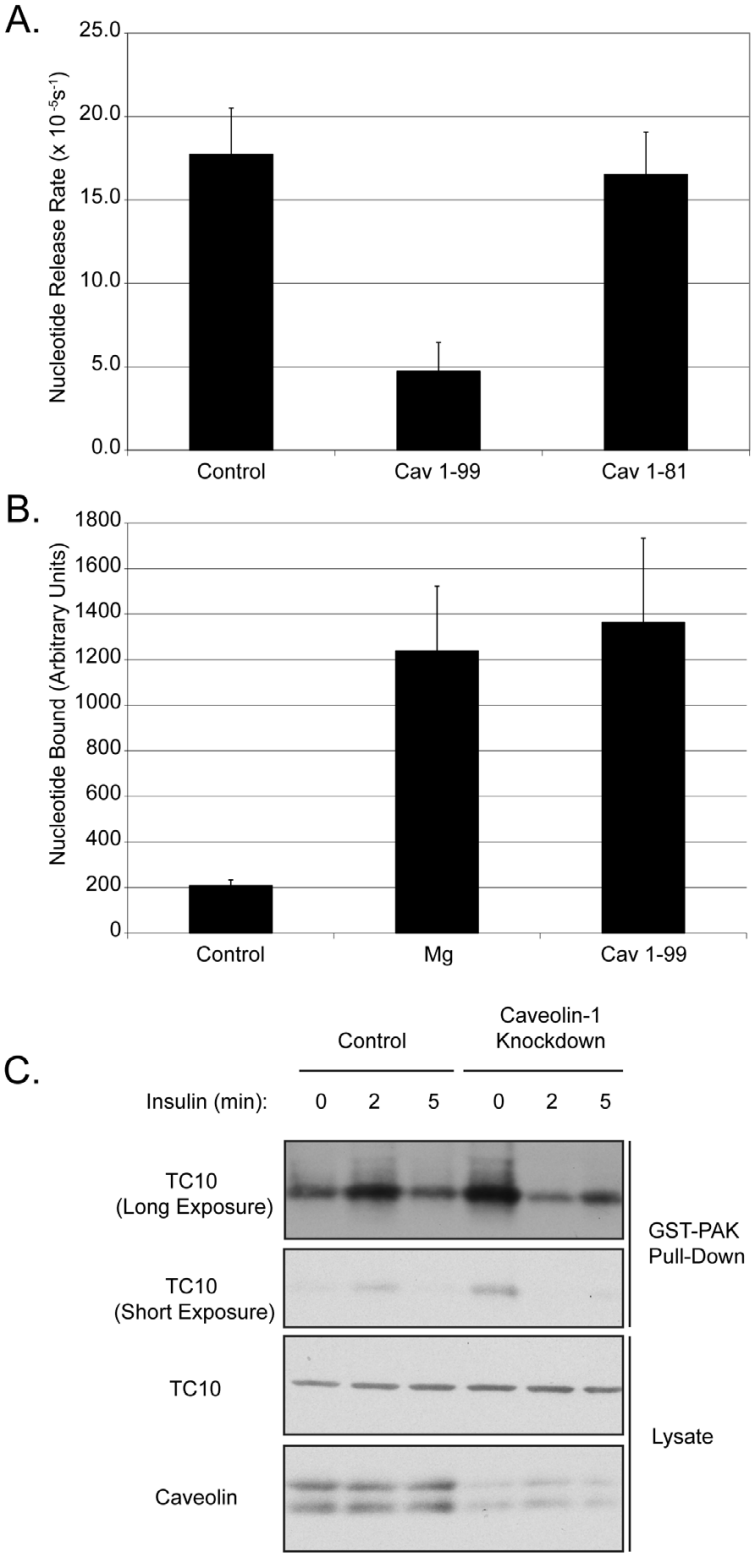
Caveolin 1 functions as a GDI for TC10. A). GST-TC10 (50 nM) was incubated with control buffer, or buffer with 10 mM MgCl_2_ or 5 mM MBP-Caveolin 1_(1–99)_ for 30 min at room temperature. GDP binding was then assayed by fluorimetry as described in Methods and Materials. B) GST-TC10-mGDP complexes were prepared as described. 50 nM of TC10 was incubated with 5 µM MBP-Caveolin or control buffer and 500 µM GTP. Fluorescence was monitored and nucleotide release rates were calculated as described in Experimental Procedures. Data for A and B are presented as mean +/− standard error (n = 3). C) Effect of Caveolin 1 knockdown on TC10 activation in 3T3-L1 adipocytes. HA-TC10 plus Control or Caveolin 1 siRNA were co-electroporated into cells for 72 h and GST-PAK pull downs were performed as described in Methods and Materials. Blots were probed with anti-HA or anti-Caveolin antibodies.

Since the binding of Caveolin 1 to TC10 is consistent with a GTPase-GDI interaction, we next tested whether Caveolin can regulate nucleotide binding on TC10. We incubated GST-TC10 with mant-GDP in either control buffer, 10 mM magnesium or a 100-fold molar excess of MBP-Caveolin1_(1–99)_. We then assayed the nucleotide binding state by fluorescence. As shown in Figure 5A, Caveolin 1 stabilizes the GDP bound state of TC10 to the same extent as 10 mM magnesium. GDI proteins function to stabilize the GDP bound state of a GTPase, so we next generated TC10-mGDP complexes and monitored the effects of MBP-Caveolin 1_(1–99)_ and MBP-Caveolin 1_(1–81)_ on the exchange rate by measuring the rate of fluorescence decrease. A 100-fold molar excess of MBP-Caveolin 1_(1–99)_, but not MBP- Caveolin 1_(1–81)_, decreased the nucleotide exchange rate by 72% (Figure 5B **)**. This suggests that Caveolin 1 can function as a GDI for TC10 *in vitro* and that this activity requires amino acids 82–99 of Caveolin 1, which includes the Caveolin scaffolding domain.

We also used siRNA in order to examine whether Caveolin can function as a physiological regulator of TC10 activation state. 3T3-L1 adipocytes were co-electroporated with HA-tagged TC10 and either control or Caveolin 1 siRNA oligonucleotides. The siRNA resulted in approximately a 90% reduction in Caveolin. We then used GST-PAK1 beads to pull-down active TC10. In control cells, TC10 activity was low, and increased by treatment of cells with insulin. Targeted knockdown of Caveolins in 3T3-L1 adipocytes resulted in greater than a 80% increase in PAK binding activity of TC10 at the basal level (Figure 5C). This suggests that Caveolin functions *in vivo* to maintain TC10 in an inactive state.

## Discussion

Magnesium is involved in stabilization of nucleotides for most GTPases [25]. GEFs activate these proteins by destabilizing the magnesium binding pocket, whereas GDIs stabilize bound magnesium [26]. Magnesium, therefore plays a key mechanistic role in all GTPases. Characterization of the magnesium-dependency of nucleotide binding *in vitro* revealed that TC10 has elevated guanine nucleotide release and exchange rates compared to other GTPases, and requires supra-physiological concentrations of magnesium to stabilize nucleotide binding.

Structural studies have shown that TC10 has a Switch I region that remains in the GDP-like conformation while GTP is bound. Data presented here indicate that the region proximal to Switch I is important for the excess magnesium requirement for binding (residues 50–82). It appears that magnesium binding may stabilize this region of the protein, in the process attenuating nucleotide exchange by TC10. These data implicate residues contacting the Switch I region of TC10 as important for both of these related phenotypes. Interestingly, both M-Ras [25] and Rac1b [26] are similarly deficient in the Switch I conformational changes, and in the case of Rac1b, results in a constitutively active form of the GTPase [26].

This analysis reveal two important points about the *in vivo* behavior of TC10 as it relates to nucleotide (and therefore effector) binding. We found that higher than physiological concentrations of magnesium are required to stabilize nucleotide binding by TC10. Therefore, this suggests that *in vivo*, a GDI may be required for regulation of TC10 activity. Reduced nucleotide affinity under physiological concentrations of magnesium suggests that nucleotides may not be stably bound in the absence of a GDI, and thus undergo rapid exchange. Since the concentrations of GTP in cells are higher than those of GDP, it is expected that TC10 would be constitutively active unless a GDI were present.

TC10 effector binding studies have revealed that wild-type TC10 binds effectors to an extent seen with the GTPase-deficient form, while the dominant-negative form cannot bind effectors [4]–[6], [8], [9]. Furthermore, overexpressed TC10 is constitutively active in both MDCK and PC12 cells, but is deactivated upon membrane translocation [19]. Together, these data suggest that TC10 is maintained in a constitutively active state *in vivo* as well as *in vitro* in the case of yeast, MDCK, PC10 and COS-1 cells, but not in 3T3-L1 adipocytes, where the G protein is inactive until cells are stimulated with insulin [3], [9].

These observations prompted us to search for a factor in 3T3-L1 cells that stabilizes nucleotide binding, leading us to identify Caveolin 1 as a GDI for TC10. Like Caveolin, TC10 is found in caveolar rosette structures in 3T3-L1 adipocytes due to the tandem acylation of the protein [1]. TC10 and Caveolin interact *in vitro* by pull-down assay, and interact with each other in cells, as shown by co-immunoprecipitation. Caveolin has been shown to interact with a wide variety of proteins, so much so that non-specific binding of TC10 to Caveolin 1 was a concern. However, sequence analyses revealed a Caveolin-binding motif in TC10; site-directed mutagenesis experiments implicated this region in the Caveolin-TC10 interaction. Moreover, the binding of TC10 to Caveolin is nucleotide-dependent and dominant-negative mutants of TC10 bind Caveolin 1 stronger than does constitutively active TC10. The same is true for GDP-bound TC10 relative to activated GTP-TC10. Data presented here also show that Caveolin 1 may function as a GDI for TC10 *in vitro*. The modulation of the TC10-Caveolin interaction by point mutants, the nucleotide dependence of this interaction, as well as the direct effect on nucleotide exchange suggests that the Caveolin-TC10 interaction is specific. Finally, knockdown of Caveolin expression in 3T3-L1 adipocytes results in higher basal activity of TC10 as measured by effector binding assay, consistent with a role of Caveolin 1 as a GDI for TC10.

Caveolin 1 has been recently implicated as a GDI for the highly similar Rho GTPase Cdc42 [19], [27]. While Caveolin has limited sequence homology to Rho and RabGDI proteins, reports have also suggested that Caveolin 1 also has potent GDI activity towards both small [19], [27]–[29] and heterotrimeric G-proteins [30], while preferentially interacting with the GDP bound state of several GTPases [19], [30], [31].

In addition to this work, studies using other Rho family GTPases, have found interactions between Caveolin 1 and Rho GTPases [32]–[34]. In the case of RhoC signaling, disruption of the Rho-Caveolin interaction via a dominant interfering Caveolin binding domain [33] or ablation of Caveolin 1 [27], [32] is associated with decreased RhoC signaling. Similarly, increased caveolin levels lead to increased RhoC signaling [32]. In this context, the small GTPase-Caveolin interaction plays a positive role, whereas in the case of TC10, Caveolin-1 appears to stabilize the inactive form of the GTPase. The differential role of Caveolin in regulation of small GTPases has been highlighted in Caveolin null MEFs, where Cdc42 and Rac1 are activated, while RhoA is inactivated [27].

One important implication of this study pertains to differences between caveolar and non-caveolar TC10. In cells (and subcellular locations) that lack Caveolin, TC10 may be maintained in a constitutively active state. In contrast, its localization in caveolae results in the association with and subsequent inhibition by Caveolin. What role caveolin binding plays in the activation of TC10 by insulin remains uncertain. Caveolin is phosphorylated on tyrosine 14 in response to insulin [35], [36]. Blocking of this phosphorylation event by overexpression of unphosphorylatable alleles of Caveolin appear to alleviate the efficiency of Caveolin to both inhibit Cdc42 [29], [37] or activate Rho [27]. It is therefore possible that this phosphorylation event may modulate the TC10-Caveolin interaction and therefore could play a role in the insulin-mediated activation of TC10. Although not examined in this manuscript, it will be interesting to see the effects of Caveolin phosphorylation or disruption on TC10 dependent insulin signaling pathways.

Spatial compartmentalization is now recognized as an important aspect of biochemical regulation, whether through proximity to regulatory proteins or through co-localization with effectors. The stabilization of TC10 by Caveolin 1 may be of importance to the spatio-temporal regulation of TC10 activity as well as its interaction with effector proteins.

## Materials and Methods

All chemicals and reagents were from Sigma-Aldrich (St. Louis, MO) unless otherwise indicated. Antibodies against HA, GST and MBP were from Santa Cruz (Santa Cruz, CA), antibodies against Caveolin were from BD Transduction Laboratories (San Jose, CA).

### Expression and Purification of Recombinant Proteins

Expression plasmids for GST-TC10 and GST-Cdc42 were obtained from Dr. Ian Macara (University of Virginia). Plasmids consisting of GST-TC10/Cdc42 chimeras were a generous gift of Dr. Wannian Yang (Geisinger Clinic, Danville, PA (18)). GST-fusion proteins were generated by induction of transformed Rosetta-gami (DE3) cells (Novagen, Madison, WI) with 10 µM IPTG overnight. Cells were collected, resuspended in PBS pH 7.4 (Invitrogen, Carlsbad, CA) with protease inhibitors (Roche Applied Sciences, Madison, WI) and French pressed twice at 15,000 psi. Lysates were clarified by centrifugation at 20,000×*g* for 30 min and then loaded onto glutathione-agarose (GE Healthcare, Waukesha, WI) for 1 h at 4°C. Proteins were then washed extensively with PBS and eluted with 50 mM glutathione in PBS. Proteins were dialysed into PBS with 50% glycerol and quantified by absorbance at 280 nm.

MBP-Caveolin 1 fusions (1–99 and 1–81) was generated by the Life Science Institute’s High Throughput Protein Core at the University of Michigan. Constructs generated by ligation-independent cloning into the pET30aMBP-LIC-TEV vector and transformed into Rosetta-gami (DE3) cells. 1L of cells were induced with 250 µM IPTG for 2h at 37°C, centrifuged and resuspended in 30 mL of MBP purification buffer (20 mM Tris pH 7.5, 200 mM NaCl, 1 mM EDTA and 0.07% beta-mercaptoethanol). Cells were lysed by sonication, clarified by centrifugation for 30 min at 9000×*g* and loaded onto 5 mL of amylose resin (New England Biolabs) for 2 h at 4°C. The slurry was then poured into a column, washed with 100 mL of MBP purification buffer and eluted with 10 mM maltose. Proteins were quantified by absorbance at 280 nM.

### Co-immunoprecipitation and Pull-Down of Proteins

For co-purification of proteins, the indicated cells were lysed by scraping into HNTG buffer (50 mM HEPES pH 7.4, 150 mM NaCl, 10% Glycerol, 1% Triton-X100 and protease inhibitors from Roche). The lysates were incubated for 30 min end over end at 4C then clarified by centrifugation at >13000 g for 15 min. Lysates were then combined with either recombinant protein bound to agarose beads or with antibodies as indicated. For immunoprecipitations, 25 µL of Protein A/G beads (Santa Cruz Biotechnology, Santa Cruz, CA) were also added. After incubation for 30 min-1 h, precipitates were washed 3–5 times with HNG buffer (HNTG buffer without Triton X-100) and resuspended in SDS sample buffer.

### Cell Culture and DNA Transfection

COS-1 cells were grown under standard conditions and were obtained from the ATCC. Transfections were performed by combining 100 ng plasmid DNA with 5.1 µL of a DMEM/Fugene mixture, prepared according to manufacturer’s instructions (Roche Applied Sciences, Indianapolis, IN). The DNA/Fugene mixture was incubated with freshly passaged cells for ∼18 h prior to collection. Mammalian expression plasmids pKH3-TC10 (*2*) and pKMyc-Caveolin 1 (19) were described previously. Point mutations of TC10 (Y37A and Y46A) were generated using Quickchange mutagenesis kit (Stratagene, La Jolla, CA) according to manufacturers instructions.

3T3-L1 cells were obtained, passaged, differentiated and electroporated as previously described (17). For Caveolin knockdown experiments 1 nmol/well of Caveolin or scrambled Stealth siRNA (Invitrogen, Carlsbad, CA) was co-electroporated with 20 µg/well pKH3-TC10 and plated in a six-well dish. Cells were re-fed at approximately 24 h intervals for 72 h.

### Nucleotide Binding and Exchange Assays

Fluorescent nucleotide binding assays were performed by combining 500 nM mantGDP (Molecular Probes, Eugene, OR) and 1 µM GST-TC10 or GST-Cdc42 in Hepes Buffered Saline (HBS; 25 mM HEPES pH 7.4, 150 mM NaCl with supplemental MgCl_2_ where indicated) and monitored at 30°C using a Fluostar Optima plate reader with appropriate filter sets (BMG Labtech, Durham, NC). Data was fitted using an exponential curve rising to a maximum using SigmaPlot (Systat, Point Richmond, CA) to determine rates. For steady state binding experiments, 50 nM mantGDP was titrated with varying concentrations of GST-TC10 or GST-Cdc42 in HBS and incubated for 1 h at room temperature. Dissociation constants were calculated according to Ahmadian *et al.* (20). Fluorescent nucleotide release experiments were conducted by incubating 1 µM protein with a 25-fold molar excess of mantGDP for 2 h at room temperature in HBS with 10 mM MgCl_2_. Excess nucleotide and metals were removed by passing protein-nucleotide complexes through a PD-10 gel filtration column (GE Healthcare, Waukesha, WI). Protein-mantGDP complexes were then combined with buffer containing indicated concentrations of Mg. Fluorescence was monitored as described above with dissociation rates calculated by fitting of data with an exponential decay curve. For nucleotide exchange data, the nucleotide release rate (k_obs_) is equal to:
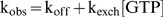



Therefore, a plot of k_obs_ vs the concentration of GTP has a slope equal to k_exch_ and an intercept equal to k_off_. These values were calculated via linear regression analysis of this data. Binding rates (k_on_) were calculated from the K_D_ and k_off_ values.

Radioactive nucleotide binding and release assays were performed as follows: 1 µg of GST-fusion protein coupled to glutathione-agarose was equilibrated in 200 µl binding buffer (25 mM Tris, pH 7.5, 40 mM NaCl, 1.0 mM DTT, and 0.5% NP-40) containing the indicated concentration of MgCl_2_ (0–100 mM), and incubated at room temperature for 10 min. 0.2 pMol [^35^S]GTPγS at a specific activity of 1000 cpm/fMol was diluted in 50 µl binding buffer plus the indicated MgCl_2_ concentration, and was added to the beads. The samples were incubated with constant inversion for 1 h. The beads were then washed three times with 1.0 ml binding buffer containing the corresponding MgCl_2_ concentration. Washed beads were re-suspended in 100 µl binding buffer and added to 5 ml scintillation cocktail for counting using a Beckman Coulter LS-6500 multi-purpose scintillation counter. GST was included in the assay as a negative control to confirm specificity of GTP binding to the GST-fusion protein.

*In vivo* TC10 activation state was determined by GST-PAK pull-down assay as described previously [17].
